# Supplementary material for: Limited Stress Response to Transplantation in the Mediterranean Macroalga Ericaria amentacea, a Key Species for Marine Forest Restoration
Source: Int J Environ Res Public Health. 2022 Sep 27;19(19):12253. doi: 10.3390/ijerph191912253 (PMC9566098; doi:10.3390/ijerph191912253)
Supplement: Supplementary file 1 [file ijerph-19-12253-s001.zip › ijerph-1896410-supplementary.pdf]

Table S1: Results of PERMANOVA (a) main test and (b) pairwise tests testing for differences in concentration of Total Phenolic Compounds (TPC) in *Ericaria amentacea* for the factors Treatment (Natural and Transplanted), Time (T0, T1, T2, T3 and T4) and Site (Barcarello and Capo Gallo). Significant p values are highlighted in bold.

**Total Phenolic Compounds (TPC)**				
**a) Main Test**				
**Source of variation**	**df**	**MS**	**Pseudo-F**	**P(perm)**
Treatment (Tr)	1	27.23	133.62	**0.053**
Time (Ti)	4	48.34	2.41	0.255
Site (Si)	1	64.59	22.42	**0.001**
Tr x Ti	3	0.54	0.65	0.626
Tr x Si	1	0.20	0.07	0.777
Ti x Si	3	19.32	6.71	**0.001**
Tr x Ti x Si	2	0.83	0.29	0.772
Residuals	64	2.88		

**b) Pair-wise tests**	**(between Times within Sites)**			
	**Barcarello**		**Capo Gallo**	
**Times**	**t**	**P(perm)**	**t**	**P(perm)**
T0, T1	1.51	0.131	2.15	**0.060**
T0, T2	0.98	0.343	4.13	**0.002**
T0, T3	1.39	0.181	9.94	**0.001**
T0, T4	5.33	**0.001**	-	
T1, T2	0.73	0.474	3.07	**0.007**
T1, T3	1.05	0.338	6.47	**0.001**
T1, T4	5.85	**0.001**	-	
T2, T3	0.20	0.841	3.49	**0.003**
T2, T4	5.37	**0.001**	-	
T3, T4	7.78	**0.001**	-	

Table S2: Results of PERMANOVA (a) main test and (b) pairwise tests testing for differences in concentration of Total Lipids (TL) in *Ericaria amentacea* for the factors Treatment (Natural and Transplanted), Time (T0, T1, T2, T3 and T4) and Site (Barcarello and Capo Gallo). Significant p values are highlighted in bold.

	**Total Lipids (TL)**			
**a) Main Test**				
**Source of variation**	**df**	**MS**	**Pseudo-F**	**P(perm)**
Treatment (Tr)	1	321.55	2.78	0.287
Time (Ti)	4	95.12	1.43	0.466
Site (Si)	1	10.36	0.37	0.553
Tr x Ti	3	6.07	4.99	0.285
Tr x Si	1	118.27	4.27	**0.046**
Ti x Si	2	62.02	2.24	0.112
Tr x Ti x Si	1	0.44	0.02	0.899
Residuals	55	27.68		

**b) Pair-wise tests**	**(between Treatments within Sites)**			
	**Barcarello**		**Capo Gallo**	
**Treatments**	**t**	**P(perm)**	**t**	**P(perm)**
NAT, TRANSP	2.06	**0.045**	3.08	**0.006**

Table S3: Results of PERMANOVA (a) main test and (b) pairwise tests testing for differences in Fatty Acid (FA) profiles in *Ericaria amentacea* for the factors Treatment (Natural and Transplanted), Time (T0, T1, T2, T3 and T4) and Site (Barcarello and Capo Gallo). Significant p values are highlighted in bold.

Fatty acids (FA)				
**a) Main Test**				
**Source of variation**	**df**	**MS**	**Pseudo-F**	**P(perm)**
Treatment (Tr)	1	9.9E-03	5.05	**0.023**
Time (Ti)	4	4.3E-02	16.18	**0.003**
Site (Si)	1	1.1E-02	12.57	**0.001**
Tr x Ti	3	4.2E-03	1.45	0.390
Tr x Si	1	2.0E-03	2.22	0.061
Ti x Si	2	2.4E-03	2.63	**0.014**
Tr x Ti x Si	1	3.0E-03	3.31	**0.016**
Residuals	54	9.0E-04		

**b) Pair-wise tests**	**(between Times within Sites and Treatments)**			
**Site: Barcarello**	**Natural**		**Transplanted**	
**Times**	**t**	**P(perm)**	**t**	**P(perm)**
T0, T1	4.98	**0.009**	3.96	**0.005**
T0, T2	2.54	**0.005**	2.81	**0.006**
T0, T3	3.58	**0.010**	4.18	**0.009**
T0, T4	4.14	**0.007**	4.18	**0.012**
T1, T2	5.25	**0.006**	4.04	**0.008**
T1, T3	6.43	**0.014**	7.83	**0.007**
T1, T4	8.05	**0.010**	7.78	**0.005**
T2, T3	2.17	**0.011**	5.22	**0.013**
T2, T4	3.60	**0.012**	6.14	**0.006**
T3, T4	2.94	**0.015**	4.71	**0.006**

**Site: Capo Gallo**	**Natural**		**Transplanted**	
**Times**	**t**	**P(perm)**	**t**	**P(perm)**
T0, T1	3.82	**0.006**	2.31	**0.024**
T0, T2	1.64	0.089	2.98	**0.008**
T1, T2	4.01	**0.007**	4.23	**0.007**

**c) Pair-wise tests**	**(between Treatments within Sites and Times)**		
**Site: Barcarello**			
**Treatments**	**Times**	**t**	**P(perm)**
NAT, TRANSP	T1	1.50	0.101
	T2	1.19	0.244
	T3	3.31	**0.013**
	T4	2.60	**0.020**

**Site: Capo Gallo**			
**Treatments**	**Times**	**t**	**P(perm)**
NAT, TRANSP	T1	2.65	**0.008**
NAT, TRANSP	T2	2.52	**0.008**

Table S4: Results of PERMANOVA (a) main test and (b) pairwise tests testing for differences in relative abundance of Fatty Acid (FA) classes in *Ericaria amentacea* for the factors Treatment (Natural and Transplanted), Time (T0, T1, T2, T3 and T4) and Site (Barcarello and Capo Gallo). Significant p values are highlighted in bold.

**Fatty acid (FA) classes**										
**a) Main Test**		**i) Saturated FA (SFA)**			**ii) Monounsaturated FA (MUFA)**			**iii) Polyunsaturated FA (PUFA)**		
**Source of variation**	**df**	**MS**	**Pseudo-F**	**P(perm)**	**MS**	**Pseudo-F**	**P(perm)**	**MS**	**Pseudo-F**	**P(perm)**
Treatment (Tr)	1	4.2E-03	3.46	0.244	9.8E-04	2.32	0.315	1.0E-02	2.58	0.291
Time (Ti)	4	9.6E-03	12.07	0.081	1.5E-02	9.76	0.097	5.4E-02	14.48	0.072
Site (Si)	1	4.7E-03	26.51	**0.001**	1.1E-03	3.66	0.064	1.4E-02	18.13	0.001
Tr x Ti	3	1.9E-03	4.48	0.293	5.0E-04	0.31	0.800	1.3E-03	0.29	0.855
Tr x Si	1	1.2E-03	6.93	**0.010**	4.3E-04	1.43	0.221	4.2E-03	5.50	**0.019**
Ti x Si	2	7.1E-04	3.97	**0.033**	1.3E-03	4.51	**0.012**	3.3E-03	4.38	**0.014**
Tr x Ti x Si	1	4.4E-04	2.44	0.132	1.7E-03	5.56	**0.022**	4.6E-03	6.07	**0.010**
Residuals	54	1.8E-04			3.0E-04			7.6E-04		
		**iv) ω-3 PUFA**			**v) ω-6 PUFA**			**vi) Bacterial FA (BAFA)**		
**Source of variation**	**df**	**MS**	**Pseudo-F**	**P(perm)**	**MS**	**Pseudo-F**	**P(perm)**	**MS**	**Pseudo-F**	**P(perm)**
Treatment (Tr)	1	1.6E-02	4.58	0.188	1.2E-03	105.98	**0.004**	2.0E-05	3.58	0.260
Time (Ti)	4	5.8E-02	28.19	**0.044**	8.5E-03	14.70	**0.046**	2.6E-04	71.44	**0.015**
Site (Si)	1	1.4E-02	22.80	**0.001**	2.6E-04	1.23	0.278	3.1E-06	0.48	0.497
Tr x Ti	3	1.6E-03	0.82	0.636	3.5E-04	1.47	0.544	9.7E-06	3.68	0.327
Tr x Si	1	3.7E-03	5.84	**0.018**	5.3E-06	0.02	0.869	5.7E-06	0.88	0.403
Ti x Si	2	1.8E-03	2.91	**0.040**	5.2E-04	2.45	0.087	3.2E-06	0.51	0.642
Tr x Ti x Si	1	2.0E-03	3.24	0.079	2.4E-04	1.13	0.284	2.5E-06	0.39	0.575
Residuals	54	6.3E-04			2.1E-04			6.4E-06		

b) Pair-wise tests

**Between Treatments within Sites**	**SFA**				**ω-3 PUFA**			
	**Barcarello**		**Capo Gallo**		**Barcarello**		**Capo Gallo**	
Treatments	**t**	**P(perm)**	**t**	**P(perm)**	**t**	**P(perm)**	**t**	**P(perm)**
NAT, TRANSP	2.28	**0.031**	4.45	**0.001**	2.95	**0.006**	3.71	**0.004**
**Between Times within Sites**	**Barcarello**		**Capo Gallo**		**Barcarello**		**Capo Gallo**	
Times	**t**	**P(perm)**	**t**	**P(perm)**	**t**	**P(perm)**	**t**	**P(perm)**
T0, T1	6.59	**0.002**	2.24	**0.045**	8.66	**0.001**	4.83	**0.003**
T0, T2	1.75	0.119	0.81	0.457	2.19	0.057	1.09	0.296
T0, T3	0.13	0.891	-		2.15	0.073	-	

T0, T4	1.91	0.079	-		2.23	**0.047**	-	
T1, T2	9.54	**0.001**	8.13	**0.001**	10.57	**0.001**	8.09	**0.001**
T1, T3	15.62	**0.001**	-		15.31	**0.001**	-	
T1, T4	10.60	**0.001**	-		20.25	**0.001**	-	
T2, T3	7.43	**0.001**	-		4.60	**0.003**	-	
T2, T4	4.06	**0.002**	-		10.80	**0.001**	-	
T3, T4	1.62	0.113	-		6.97	**0.001**	-	

**Between Times within Sites and Treatments**	**MUFA**				**PUFA**			
	**Natural**		**Transplanted**		**Natural**		**Transplanted**	
**Site: Barcarello**	**t**	**P(perm)**	**t**	**P(perm)**	**t**	**P(perm)**	**t**	**P(perm)**
T0, T1	1.52	0.171	0.78	0.425	3.40	0.010	2.54	0.074
T0, T2	1.82	0.116	0.68	0.461	0.91	0.403	0.15	0.853
T0, T3	2.12	0.093	1.99	0.094	1.64	0.205	3.29	**0.021**
T0, T4	4.12	**0.006**	5.15	**0.008**	4.08	**0.012**	3.48	**0.027**
T1, T2	7.73	**0.012**	2.56	**0.047**	9.51	**0.009**	5.06	**0.009**
T1, T3	9.36	**0.021**	4.27	**0.008**	12.66	**0.008**	10.98	**0.007**
T1, T4	9.38	**0.009**	8.63	**0.011**	16.27	**0.014**	9.04	**0.010**
T2, T3	0.53	0.563	2.93	**0.024**	1.79	0.116	7.30	**0.010**
T2, T4	3.85	**0.018**	9.24	**0.006**	7.00	**0.006**	6.14	**0.007**
T3, T4	3.62	**0.012**	5.83	**0.010**	6.28	**0.006**	1.48	0.162
**Site: Capo Gallo**	**t**	**P(perm)**	**t**	**P(perm)**	**t**	**P(perm)**	**t**	**P(perm)**
T0, T1	0.44	0.710	0.01	1.000	1.31	0.269	0.09	0.877
T0, T2	0.02	0.994	2.59	0.055	0.66	0.560	4.81	**0.010**
T1, T2	0.40	0.684	1.95	0.081	2.67	**0.027**	5.25	**0.007**

**Between Treatments within Sites and Times**		**Barcarello**		**Capo Gallo**		**Barcarello**		**Capo Gallo**	
		**t**	**P(perm)**	**t**	**P(perm)**	**t**	**P(perm)**	**t**	**P(perm)**
NAT, TRANSP	T1	1.06	0.299	0.27	0.860	1.31	0.235	1.26	0.260
NAT, TRANSP	T2	2.66	**0.024**	2.45	0.078	1.69	0.120	5.04	**0.006**
NAT, TRANSP	T3	0.14	0.841	-		4.59	**0.009**	-	
NAT, TRANSP	T4	1.69	0.118	-		0.19	0.836	-	

**Between Times**	**ω-6 PUFA**		**BAFA**	
	**t**	**P(perm)**	**t**	**P(perm)**
T0, T1	123.40	**0.006**	2.59	0.234
T0, T2	3.71	0.167	6.00	0.098
T0, T3	7.35	**0.001**	6.28	**0.001**

T0, T4	4.58	**0.002**	6.36	**0.001**
T1, T2	2.48	0.259	8.32	0.077
T1, T3	0.85	0.425	12.95	**0.001**
T1, T4	2.42	**0.027**	11.18	**0.001**
T2, T3	1.92	0.061	1.75	0.092
T2, T4	0.20	0.839	2.54	**0.020**
T3, T4	1.78	0.088	1.10	0.297

**Between treatments**	**t**	**P(perm)**
NAT, TRANSP	10.30	**0.004**
